# Effects of maternal antibodies against myostatin on post-hatch chicken growth and muscle mass in Sentul Indonesian indigenous chicken

**DOI:** 10.14202/vetworld.2025.388-396

**Published:** 2025-02-19

**Authors:** Sri Murtini, Asep Gunawan, Isyana Khaerunnisa, Dwi Lestari, Rajma Fastawa, Anneke Anggraeni, Yong Soo Kim, Cece Sumantri

**Affiliations:** 1 Division of Medical Microbiology, School of Veterinary Medicine and Biomedical Science, IPB University, Bogor, 16680, Indonesia; 2 Department of Animal Production and Technology, Faculty of Animal Science, IPB University, Bogor, 16680, Indonesia; 3 Research Center for Applied Zoology, National Research and Innovation Agency, Bogor, 16911, Indonesia; 4 Department of Animal Husbandry and Fisheries, Sidenreng Rappang, 91611, Indonesia; 5 Research Center for Animal Husbandry, National Research and Innovation Agency, Bogor, 16911, Indonesia; 6 Department of Human Nutrition, Food, and Animal Science, College of Tropical Agriculture and Human Resources, University of Hawaii at Manoa, Honolulu, 96822, United States

**Keywords:** carcass traits, maternal immunization, muscle growth, myostatin, Sentul chicken

## Abstract

**Background and Aim:**

Myostatin (MSTN) is a negative regulator of skeletal muscle growth, and its suppression could enhance muscle mass. This study investigated the effects of maternal immunization against MSTN on post-hatch growth, carcass characteristics, and muscle fiber size in Sentul Indonesian indigenous chickens.

**Materials and Methods:**

Seventy-five: Sentul hens were divided into three groups: Control (CON), KLH-immunized (KLH), and MSTN-conjugated KLH immunized (KLH-MSTN). The hens were immunized at 6 months, with boosters at 3 and 6 weeks after initial immunization. Serum and egg yolk antibody titers were measured through enzyme-linked immunosorbent assay. Offspring growth and carcass traits were evaluated at 12 weeks. Histological muscle fiber analysis was performed using ImageJ. Data were analyzed using a one-way analysis of variance and Tukey Honest significant difference tests.

**Results:**

Anti-MSTN antibodies were detected in 60% of KLH-MSTN hens 3 weeks post-immunization but declined to 10–30% in later collections. Male offspring in the KLH-MSTN and KLH groups exhibited significantly higher carcass, thigh, and drumstick weights than the CON group, although muscle weights showed no significant differences. In females, only thigh muscle weight in the KLH-MSTN group was significantly higher than in the CON group. Muscle fiber diameters in all measured muscles were significantly larger in the KLH-MSTN group compared to the CON and KLH groups.

**Conclusion:**

Maternal immunization with KLH-MSTN increased muscle fiber size but did not significantly enhance overall muscle weight in Sentul chicken offspring, except for the thigh muscle in females. This suggests that MSTN immunization may have limited utility in enhancing muscle growth in this chicken breed.

## INTRODUCTION

Myostatin (MSTN), a transforming growth factor-β (TGF-β) superfamily member, is a negative regulator of skeletal muscle growth. In addition, many studies have shown that MSTN suppression enhanced muscle mass under normal or pathologic conditions that induce muscle loss in animals [1–6], indicating that suppressing the biological activity of MSTN, either during embryonic development or postnatal growth, would be an effective strategy to improve skeletal muscle growth and carcass composition in farm animals.

MSTN is also involved in several muscle diseases and is a potential therapeutic target [7–9]. It has been demonstrated that in-ovo administration of anti-MSTN antibodies increases post-hatch growth of skeletal muscles [7], indicating that blocking the biological activity of MSTN during embryonic development is an effective strategy to improve chicken growth. Kim *et al*. [8] found that anti-MSTN injection in egg yolk produced heavier chicks (4.2%) and higher muscle mass (5.5%) than control eggs that were not injected with anti-MSTN. Abdel-Gawad *et al*. [9] also found that anti-myostatin injection in Fayoumi chicken eggs resulted in an increase in body weight and body weight gain compared with controls that were only injected with saline. In addition, the feed conversion ratio was significantly lower than that of the control.

To the best of our knowledge, the use of anti-MSTN antibodies in chicken, especially in Indonesia, has never been done. Research related to MSTN in local Indonesian chickens has been conducted regarding the diversity of mstn genes in local Indonesian chickens [10, 11] and their relationship with production traits [12], but the use of anti-MSTN has never been performed in local Indonesian chickens. The use of anti-MSTN in an effort to increase chicken muscle growth in Indonesia is one promising way. Improving muscle growth in Indonesian indigenous chickens is important for increasing their economic value. Therefore, this study aimed to evaluate the effects of maternal immunization with a myostatin peptide conjugated to keyhole limpet hemocyanin (KLH)-MSTN on the transfer of anti-MSTN antibodies to offspring and their subsequent impact on post-hatch growth, carcass traits, and muscle fiber characteristics in Sentul Indonesian indigenous chickens.

## MATERIALS AND METHODS

### Ethical approval

All procedures in this study were approved by the Animal Ethics Committee of IPB University (Approval No.: 94-2018IPB).

### Study period and location

This study was conducted from January to June 2019. Sentul chickens were maintained in the field laboratory of the Faculty of Animal Science, IPB University. Muscle size measurements were performed in the Pathology Laboratory, School of Veterinary Medicine and Biomedical Science, IPB University. Antibody titer measurements were performed in the Immunology Laboratory, School of Veterinary Medicine and Biomedical Science, IPB University.

### Antigen preparation

An MSTN peptide fragment (VFLQKYPHTHLVHQA), representing amino acid sequences from 50 to 64 of the active form of MSTN, was commercially synthesized and conjugated to KLH (Global Peptide Services, Co. USA). A cysteine-glycine sequence was inserted at the N-terminal side of the peptides to form a thioether linkage during the generation of KLH-MSTN peptide conjugates. Succinimidyl 4-(N-maleimidomethyl)cycholexane-1-carboxy-(6-amidocaproate) (Pierce, Rockford, IL), a sulfhydryl- and amine-reactive heterobifunctional cross-linking agent, was used to conjugate peptides to the carrier protein. The KLH-MSTN protein was used as an antigen since a previous study demonstrated the production of MSTN-binding antibodies by subcutaneous injection of KLH-MSTN in female mice [13].

### Animals and immunization

Acclimatization was carried out for seven days before the experiment. Seventy-five Sentul hens (6 months old) were divided into three groups of 25 birds per group: Control (CON), KLH, and KLH-MSTN. The hens were vaccinated with the ND vaccine (Medivac ND Hitchner B1) at 3 and 6 months of age. The CON group received no immunization, and the KLH group received 1 mg of KLH and 1 mg of KLH-MSTN intramuscularly. Antigens were prepared in 500 µL of phosphate-buffered saline (PBS) and emulsified with the same amount of complete Freund’s adjuvant or incomplete Freund’s adjuvant (500 mL) for the first immunization and booster, respectively. The first immunization was performed when the hens reached 6 months old (the age of the first laying was around 7 months old). Booster immunization was administered at 3 and 6 weeks after the first booster. Blood samples (1 mL) were collected from the brachial vein at each booster time and every 2 weeks for 5 months after the last booster immunization. Aliquots of antisera were prepared and stored at –20°C for later analysis.

Hens (16 weeks old) in each group were housed with five roosters (8 months old) in wire cages at a temperature of around 24°C. The Roosters in each group were rotated weekly among groups. Eggs were collected daily, and 4 were taken weekly from each treatment for 3 consecutive weeks.

### Egg incubation and post-hatch animal care

Eggs were collected and stored at room temperature (25°C) and placed into an incubation machine for hatching every 3 days. The production of anti-MSTN antibodies was examined in serum and egg yolks. The remaining eggs were hatched, and chicks were raised until 12 and 20 weeks. The starter diet (1 day-3 weeks old) contained 4,080 kcal/kg energy and 19.03% crude protein; the grower diet (start at 3 weeks) contained 4,001 kcal/kg and 17.42% crude protein. Drinking water is provided *ad libitum*. Chicks were vaccinated with the ND vaccine (Medivac ND La Sota) on day 4 and week 4. Blood was collected for ND antibody and anti-MSTN antibody titers on day 4 (before vaccination), 2 and 4 weeks after the first and second vaccination, and parasite infections were evaluated monthly. Chickens were sacrificed at 12 weeks of age to collect carcass data. Carcass data included live weight, carcass weight, commercial cuts (breast, thighs, drumsticks, and wings), muscle (breast muscle, thigh muscle, and drumstick muscle), and percentage of carcass, commercial cuts, and muscles. Muscle histological characteristics were also examined. All experiments were conducted in accordance with the animal ethics guidelines.

### Carcass measurement

Chickens were weighed before slaughter as live weight, then sacrificed and weighed upon bleeding as slaughter weight. Feet, shanks, head, and neck were removed, and carcasses were weighed immediately. Carcasses were dissected to obtain various body parts (breast, back, thighs, wings, drumstick, *pectoralis* *major* muscle, and *pectoralis* *minor* muscle). The carcass percentage was calculated as the ratio of carcass to live weight. The percentages of breasts, thighs, back, wings, and drumsticks were calculated based on the ratio of the weight of the cut parts to the carcass weight. The percentage of muscle weight was calculated based on the ratio of muscle weight to carcass weight.

### Immunoglobulin Y (IgY) preparation from egg yolk and serum

Egg yolk IgY was prepared as described by Jin *et al*. [14]. Egg yolk was separated from egg whites and diluted with 9 volumes of acidified distilled water (pH 5.0). The acidified solution was frozen overnight at –20°C, then defrosted and centrifuged at 10,000× *g* for 30 min. The supernatant was collected, and IgY was precipitated by adding 50% ammonium sulfate. Ammonium sulfate-precipitated IgY was collected through centrifugation at 1,000× *g* for 15 min and diluted in 1.5 mL PBS. The titer was measured using a 100-fold diluted IgY solution.

Blood serum was collected by separating the serum portion from the whole blood using a centrifuge until the serum was separated. The sample was then diluted (1:100) with PBS. Then, the serum was stored for measurement of antibody titers using the indirect enzyme-linked immunosorbent assay (ELISA) method.

### Antibody titer

The antibody titers against KLH and MSTN were measured using the indirect ELISA method [15]. KLH and MSTN were used as coating antigens at a concentration of 1.2 mg/mL. Coating antigens (100 µL) were added to the wells of a 96-well microplate and incubated at 4°C for 12 h. The microplate was washed 4 times with PBS with 0.1% Tween 20, pH 7.4 solution (PBST-20), blocked with 100 µL of 0.3% bovine serum albumin (A9418, Sigma Aldrich, Darmstadt, Germany) in addition to each well, and incubated for 60 min at 4°C. The microplates were then washed 4 times using PBST-20.

The diluted samples (100 µL) were added to each well of a microplate and incubated for 60 min at 37°C. The microplate was then washed again 3 times. 100 µL anti-chicken IgY conjugated to horseradish peroxidase solution (A9046-Sigma Aldrich) was added into each well and incubated for 30 min at 25°C. Next, 100 µL of tetramethylbenzidine substrate (T0440 Sigma-Aldrich) was added and incubated for 15 min at 25°C, followed by the addition of 100 µL of stop solution (RABSTOP3, Sigma Aldrich). The absorbance at 630 nm was measured using a microplate reader (Bio-Rad, USA). The cutoff value was calculated based on the positive standard value added to the standard deviation. Samples were categorized as positive if the absorbance value exceeded the cutoff value.

### Muscle fiber size measurement

**Figure 1:**
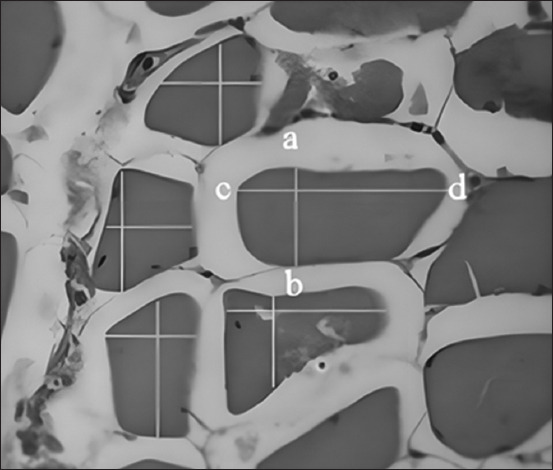
Muscle fiber diameter measurement. The height (from a to b) and width (from c to d) were measured, and the fiber diameter was estimated from the average length and width.

The samples were 12-week-old Sentul chicken. The muscle parts of the thigh, drumstick, lower breast, and upper breast were removed and separated from the carcass. The samples were then fixed in buffered neutral formalin (PBS and formalin 10%) solution for at least 3 × 24 h. The samples were then trimmed to select muscle parts, inserted into a tissue cassette, and labeled. The samples were then dehydrated in a graded alcohol series (70%–100%), cleared in xylol, and impregnated in paraffin. Cross-sections (4–5 µm) of the muscles were cut using a microtome. The sections were carefully placed on the surface of the water in a water bath at 46**°**C, placed onto microscope slides, placed into an incubator at 60**°**C, then stained with hematoxylin and eosin dye, and covered with cover glasses. The staining results were then viewed under a microscope. The stained cells were then examined under a microscope (Olympus CX23, Japan) and captured for further analysis using ImageJ software (National Institute of Health, USA). The diameter of each cell was measured using ImageJ software on a micrometer (µm) or micron unit. Measurements were performed by measuring the height (from a to b) and width (from c to d) (Figure 1) of the cell. The height and width were averaged to obtain the average cell diameter. Height and width measurements were performed in 10 fields of view with 10 random muscles in each field.

### Statistical analysis

The data were analyzed using the General Linear Model procedure in JMP 14.0 software (SAS Institute, Cary, NC, USA). All quantitative data, including carcass characteristics, muscle weights, and muscle fiber diameters, were tested for normality and homogeneity of variance before analysis. Comparisons among the treatment groups (CON, KLH, and KLH-MSTN) were conducted using analysis of variance. When significant differences were detected (p ≤ 0.05), the Tukey Honest significant difference test was applied for post-hoc pairwise comparisons. Results are presented as mean ± standard error of the mean.

## RESULTS

### Anti-MSTN antibody titer after KLH-MSTN antigen administration

In the KLH-MSTN group, 60% of the hens carried anti-MSTN antibodies in the first blood collection at 3 weeks after the first antigen administration. The percentages of hens carrying anti-MSTN antibodies in serum were very low from the second blood collection to the last blood collection, with a 10%–30% range (Table 1), indicating a gradual titer decrease after the 1^st^ immunization. The percentages of Sentul chicken egg yolk carrying anti-MSTN antibodies in the KLH-MSTN group at the 1^st^, 2^nd^, 3^rd^, and 4^th^ collections were 25%, 0%, 100%, and 0%, respectively (Table 2), showing irregular anti-MSTN presence during egg collections.

**Table 1:** Percentage of Sentul chickens containing MSTN antibody titers in blood.

Blood collection	Group		

	CON (%) (n = 10)	KLH (%) (n = 10)	KLH-MSTN (%) (n = 10)
1^st^	0	0	60
2^nd^	0	10	10
3^rd^	0	0	30
4^th^	0	30	20
5^th^	0	0	10
6^th^	0	40	20
7^th^	0	10	20

The 1^st^ and 2^nd^ blood collections were performed at the time of the 1^st^ and 2^nd^ booster immunizations, respectively. The 1^st^ and 2^nd^ immunization were performed 3 and 6 weeks after the first immunization. The 3^rd^–7^th^ week collections were collected every 2 weeks after the 2^nd^ booster. The KLH and MSTN group titers were measured against the coating antigens KLH and MSTN, respectively. The control titer used untreated chicken serum as the coating antigen, CON=Control, MSTN=Myostatin, KLH=Keyhole limpet hemocyanin

**Table 2:** Percentage of Sentul chickens containing MSTN antibody titers in the yolk.

Yolk collection	Group		

	CON (%) (n = 4)	KLH (%) (n = 4)	KLH-MSTN (%) (n = 4)
1^st^	0	0	25
2^nd^	0	0	0
3^rd^	0	50	100
4^th^	0	0	0

The KLH and MSTN group titers were measured against the coating antigens KLH and MSTN, respectively. Control titer: Untreated chicken serum, CON=Control, MSTN=Myostatin, KLH=Keyhole limpet hemocyanin

### Carcass characteristics of Sentul chicken offspring from hens immunized for KLH-MSTN antigen

The live, slaughter, and carcass weights of male Sentul chickens at 12 weeks in the KLH group were significantly heavier than those in the KLH-MSTN and CON groups, and those weights in the KLH-MSTN group were significantly heavier than those in the CON group (Table 3). The thigh and drumstick weights of male Sentul chickens at 12 weeks in the KLH and KLH-MSTN groups were significantly heavier than those in the CON group, but the *pectoralis*, thigh, and drumstick muscle weights were not significantly different among the three groups. The live, slaughter, and carcass weights of female Sentul chickens at 12 weeks were not significantly different among the three groups (Table 4). The thigh muscle weight was significantly greater in the KLH-MSTN group than in the CON group. The drumstick muscle weight was significantly greater in the KLH group than in the CON and KLH-MSTN groups.

**Table 3:** Slaughter weight, carcass weight, and carcass percentages of 12-week-old male Sentul chicken.

Traits	Group		

	CON (n = 14)	KLH (n = 9)	KLH-MSTN (n = 20)
Liver weight (g)	805.30 ± 15.80^c^	1024.2 ± 21.40^a^	943.80 ± 25.10^b^
Slaughter weight (g)	788.93 ± 14.90^c^	982.70 ± 23.20^a^	890.30 ± 26.40^b^
Carcass weight (g)	487.50 ± 10.00^c^	610.60 ± 12.80^a^	554.90 ± 14.60^b^
Breast weight (g)	122.00 ± 3.55	155.00 ± 3.68	139.45 ± 4.12
Back weight (g)	112.79 ± 2.55^b^	143.33 ± 9.05^a^	129.65 ± 4.07^a^
*Wing weight (g)	76.50 ± 1.64	93.22 ± 3.23	85.25 ± 2.12
*Thigh weight (g)	85.64 ± 2.45^b^	104.44 ± 3.51^a^	99.55 ± 2.52^a^
*Drumstick weight (g)	86.00 ± 1.80^b^	106.22 ± 3.44^a^	97.90 ± 3.42^a^
*Pectoralis major* muscle weight (g)	61.21 ± 2.07	77.67 ± 2.66	68.65 ± 1.99
*Pectoralis minor* muscle weight (g)	25.93 ± 0.99	30.11 ± 1.74	27.80 ± 0.78
*Thigh muscle weight (g)	60.39 ± 2.23	71.67 ± 3.05	69.25 ± 2.02
*Drumstick muscle weight (g)	52.21 ± 1.23	63.78 ± 1.75	59.70 ± 2.05
Carcass weight (%)	60.55 ± 0.54	59.62 ± 0.41	58.82 ± 0.38
Breast weight (%)	25.01 ± 0.44	25.39 ± 0.41	25.12 ± 0.26
Back weight (%)	23.15 ± 0.33	23.52 ± 1.40	23.33 ± 0.29
Wing weight (%)	15.71 ± 0.24	15.26 ± 0.36	15.41 ± 0.21
Thigh weight (%)	17.55 ± 0.28	17.14 ± 0.59	17.97 ± 0.20
Drumstick weight (%)	17.65 ± 0.19	17.38 ± 0.35	17.60 ± 0.26
*Pectoralis major* muscle weight (g)	12.53 ± 0.27	12.74 ± 0.41	12.37 ± 0.13
*Pectoralis minor* muscle weight (%)	5.31 ± 0.14	4.91 ± 0.19	5.03 ± 0.09
Thigh muscle weight (%)	12.38 ± 0.35	11.76 ± 0.49	12.49 ± 0.18
Drumstick muscle weight (%)	10.71 ± 0.10	10.44 ± 0.15	10.74 ± 0.18

Means not sharing the same superscript in the same row differs at the p *<* 0.05.

* Combined weight of the right and left sides, CON=Control, MSTN=Myostatin, KLH=Keyhole limpet hemocyanin

**Table 4:** Slaughter weight, carcass weight, and carcass percentages of 12-week-old female Sentul chicken.

Traits	Group		

	CON (n = 45)	KLH (n = 22)	KLH-MSTN (n = 35)
Liver weight (g)	753.40 ± 13.20	758.10 ± 25.10	749.10 ± 15.10
Slaughter weight (g)	735.10 ± 13.40	716.10 ± 28.20	712.30 ± 17.30
Carcass weight (g)	459.44 ± 7.77	454.80 ± 18.30	444.10 ± 10.40
Breast weight (g)	117.76 ± 2.40	116.30 ± 5.73	114.94 ± 3.07
Back weight (g)	105.49 ± 2.22	103.27 ± 5.08	99.83 ± 2.60
*Wings weight (g)	71.64 ± 1.34	72.00 ± 2.68	68.83 ± 1.49
*Thigh weight (g)	80.11 ± 1.53	78.50 ± 2.47	78.63 ± 1.86
*Drumstick weight (g)	80.49 ± 1.38	77.68 ± 3.07	76.03 ± 1.91
Pectoralis major muscle weight (g)	57.51 ± 1.33	56.82 ± 3.31	57.03 ± 1.69
*Pectoralis minor* muscle weight (g)	23.33 ± 0.61	22.36 ± 1.44	22.94 ± 0.74
*Thigh muscle weight (g)	59.01 ± 1.21^b^	61.59 ± 2.81^ab^	66.11 ± 2.64^a^
*Drumstick muscle weight (g)	48.97 ± 0.93^b^	51.36 ± 2.82^a^	46.86 ± 1.42^b^
Carcass weight (%)	61.16 ± 0.64	59.77 ± 0.95	59.09 ± 0.77
Breast weight (%)	25.62 ± 0.28	25.34 ± 0.53	25.97 ± 0.35
Back weight (%)	22.94 ± 0.23	22.60 ± 0.38	22.55 ± 0.25
Wing weight (%)	15.62 ± 0.17	15.92 ± 0.24	15.60 ± 0.16
Thighs weight (%)	17.43 ± 0.14	17.46 ± 0.37	17.79 ± 0.18
Drumsticks weight (%)	17.55 ± 0.16	17.16 ± 0.32	17.18 ± 0.16
*Pectoralis major* muscle weight (%)	12.51 ± 0.19	12.33 ± 0.38	12.86 ± 0.21
*Pectoralis minor* muscle weight (%)	5.07 ± 0.09	4.84 ± 0.22	5.17 ± 0.10
Thigh muscle weight (%)	12.87 ± 0.18^b^	13.83 ± 0.67^ab^	14.98 ± 0.52^a^
Drumstick muscle weight (%)	10.65 ± 0.08	11.40 ± 0.57	10.56 ± 0.18

Means not sharing the same superscript in the same row differs at the p *<* 0.05.

* Combined weight of the right and left sides, CON=Control, MSTN=Myostatin, KLH=Keyhole limpet hemocyanin

### Muscle histology

**Figure 2:**
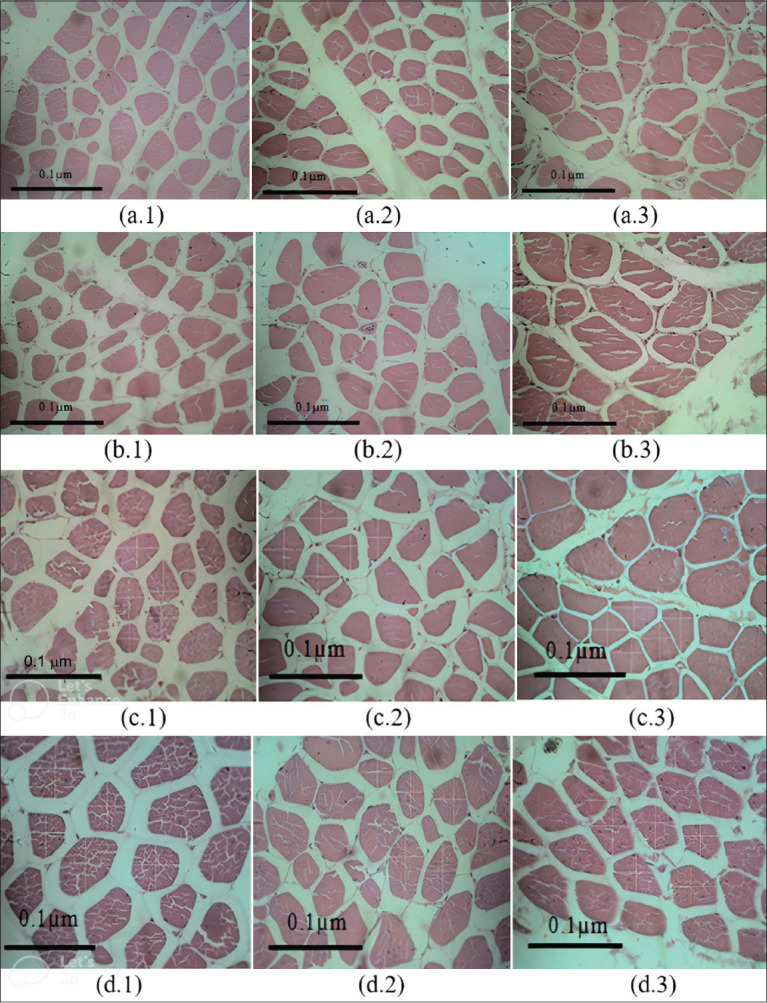
Density of muscle from groups CON (1), KLH (2), and MSTN (3) in the thigh muscle (a), drumstick muscle (b), breast muscle (upper side) (c), and breast muscle (lower side) (d) at 400× magnification with hematoxylin and eosin staining. CON=Control, MSTN=Myostatin, KLH=Keyhole limpet hemocyanin.

Microscopic observations of each group’s thigh, drumstick, and breast muscle showed no pathological changes in myofibril inflammation, degeneration, or necrosis (Figure 2). The diameters of the thigh, drumstick, and breast (upper and lower sides) muscles were significantly longer in the KLH-MSTN group than in the CON and KLH groups (Table 5).

**Table 5:** The average diameter of the thigh and breast muscle fiber cross-sections of 12-week-old chickens.

Muscle part (µm)	Group		

	CON (n = 3)	KLH (n = 3)	KLH-MSTN (n = 3)
Thigh	27.74 ± 7.65^b^	26.98 ± 7.39^b^	30.62 ± 7.47^a^
Drumstick	28.99 ± 7.78^b^	27.40 ± 7.00^b^	34.76 ± 7.16^a^
Breast, upper side	39.62 ± 10.82^b^	38.53 ± 8.80^b^	43.16 ± 8.74^a^
Breast, lower side	39.22 ± 9.93^b^	37.08 ± 8.44^b^	43.97 ± 10.25^a^

Means not sharing the same superscript in the same row differs at the p *<* 0.05. CON; Control group, KLH; KLH group, MSTN: MSTNpeptide-KLH-conjugated group, CON=Control, MSTN=Myostatin, KLH=Keyhole limpet hemocyanin

## DISCUSSION

MSTN, a member of the TGF-β superfamily, suppresses muscle mass growth [16]. The lack of MSTN results in excessive skeletal muscle growth [17], and MSTN-knockout mice exhibit a dramatic and broader increase in skeletal muscle mass [18]. The increase in skeletal muscle mass was due to an increase in the number of muscle fibers (hyperplasia) and an increase in the size of muscle fibers (hypertrophy) [19]. Anti-MSTN antibodies bind to MSTN and block the MSTN signaling pathway [20]. Numerous studies have shown that MSTN-antibodies enhance skeletal muscle growth and body mass [7–9]. Antibodies from hens are transferred through blood serum to the ovary and into egg yolk [21], and antibodies in egg yolk have the same concentration in hen serum [22]. Antibodies in the yolk are absorbed during embryo development and can be found in blood circulation [23].

Sentul hens were immunized in this study with KLH or MSTN peptide fragments (13 amino acids) conjugated to KLH (KLH-MSTN). KLH conjugation was necessary because the MSTN peptide was too small to induce immune reactions [24]. Thus, KLH was used as a carrier protein. In addition to immunization with KLH-MSTN, immunization with KLH was added to examine whether KLH-immunization affects the growth of offspring (F1). The highest MSTN titer in serum was found at 3 weeks after the first KLH-MSTN immunization. Approximately 60% of hens showed titers at 3 weeks after the immunization and 10%–30% of hens showed titers in subsequent collections, indicating a weak immune reaction. The highest serum KLH titer (40%) was lower in the KLH group than in the MSTN group, with some collections showing no titers. Unlike the current result, other similar studies [25, 26] reported that immunization with KLH-conjugated MSTN fragments produced titers against MSTN in all hens used in their study. Antibody titers are affected by various factors, including the antigen type and dose, adjuvant, route of application, inoculation frequency, and age [27]. Considering that the present study used a similar antigen, dose, and adjuvant, it is possible that the Sentul hens were much less responsive to the KLH-MSTN than other fast-growing hens. The MSTN titer was also observed in the eggs of the KLH-MSTN groups, but some collections showed no MSTN titer, as expected from the serum titer results.

Our results showed that the carcass, thigh, and drumstick weights of male offspring were significantly enhanced by the immunization of hens with KLH and KLH-MSTN, whereas the weights of the *pectoralis*, thigh, and drumstick muscles were not affected by the immunization. These results indicate that egg york had no effect on muscle growth in male offspring, but the presence of anti-MSTN improved the growth of other tissues. These results are contrary to other results reported by Moon *et al*. [25] and Mishra *et al*. [26], in which the growth rate and muscle mass of offspring from hens immunized with MSTN fragments conjugated to KLH were significantly suppressed despite the presence of anti-MSTN IgY in hen eggs. While the current results are inconsistent with the results of previous studies by [25, 26], the three results together indicate that the immunization of hens with MSTN is ineffective in improving the muscle growth of offspring. The improvement in growth of male offspring from hens immunized with KLH was unexpected because KLH is generally known not to be associated with biological activities in animals. We cannot explain how the presence of anti-KLH antibodies in egg york improved the growth of male Sentul chicken. Thus, further investigation is needed to confirm and understand the role of anti-KLH antibodies in Sentul chicken growth.

In contrast to male offspring, the carcass, thigh, and drumstick weights of female offspring in the KLH and KLH-MSTN groups were not significantly different from those in the CON group. However, the thigh muscle weight and the drumstick muscle weight were increased by KLH-MSTN and KLH-MSTN immunization, respectively. This result indicates that body and muscle growth responses to the presence of anti-KLH and anti-MSTN antibodies differ between male and female Sentul chickens. Again, we cannot explain the different body and muscle growth responses to anti-KLH and anti-MSTN antibodies between males and females. Thus, further investigation is required to confirm and understand the different responses of male and female Sentul chickens.

## CONCLUSION

This study evaluated the effects of maternal immunization with a myostatin peptide conjugated to KLH-MSTN on the growth performance, carcass characteristics, and muscle fiber morphology of Sentul Indonesian indigenous chicken offspring. Results showed that anti-myostatin antibodies were successfully generated in some immunized hens, with antibody titers detected in both serum and egg yolks. Male offspring from the KLH-MSTN and KLH groups exhibited significantly higher carcass, thigh, and drumstick weights compared to the control group, although there was no significant improvement in muscle weights. In female offspring, the thigh muscle weight was significantly increased in the KLH-MSTN group, but overall growth responses were limited. Muscle fiber diameter was larger in the KLH-MSTN group, indicating potential histological impacts on muscle structure.

This study provides novel insights into the application of maternal immunization against myostatin in Sentul chickens, a locally significant breed in Indonesia. The integration of carcass, growth, and histological analyses adds robustness to the findings, contributing to the understanding of maternal immunization effects in poultry. However, variability in antibody titers was observed with only a portion of hens maintaining detectable levels of anti-myostatin antibodies. In addition, the differences in responses between male and female offspring highlight potential sex-specific effects, which remain unexplained. The lack of significant improvements in muscle weights suggests the limited efficacy of the immunization strategy for enhancing economic traits in this breed.

Future studies should focus on optimizing immunization protocols, including antigen dose, adjuvant type, and administration timing, to enhance antibody production. Investigations into genetic factors influencing the immune response and sex-specific growth patterns in offspring are warranted. In addition, exploring alternative strategies, such as genetic or dietary interventions, could complement immunization efforts to improve muscle growth in indigenous poultry breeds.

## AUTHORS’ CONTRIBUTIONS

SM, IK, YSK, and CS: Conceptualization of the study. AG, YSK, and CS: Validation, investigation, and supervision. SM, IK, DL, RF, and AA: Methodology and data analysis. SM, DL, and RF: Data visualization and interpretation. SM, IK, and DL: Drafted the manuscript. AA, YSK, CS, and AG: Edited the manuscript. All authors have read and approved the final manuscript.
